# ‘Hotspots’ of Antigen Presentation Revealed by Human Leukocyte Antigen Ligandomics for Neoantigen Prioritization

**DOI:** 10.3389/fimmu.2017.01367

**Published:** 2017-10-20

**Authors:** Markus Müller, David Gfeller, George Coukos, Michal Bassani-Sternberg

**Affiliations:** ^1^Vital-IT, Swiss Institute of Bioinformatics, Lausanne, Switzerland; ^2^Swiss Institute of Bioinformatics, Lausanne, Switzerland; ^3^Ludwig Cancer Research Center, University of Lausanne, Epalinges, Switzerland; ^4^Department of Oncology, Lausanne University Hospital, Lausanne, Switzerland

**Keywords:** mass spectrometry, immunopeptidomics, antigen processing and presentation, human leukocyte antigen-binding prediction, neoantigens, cancer immunotherapy, personalized cancer vaccines

## Abstract

The remarkable clinical efficacy of the immune checkpoint blockade therapies has motivated researchers to discover immunogenic epitopes and exploit them for personalized vaccines. Human leukocyte antigen (HLA)-binding peptides derived from processing and presentation of mutated proteins are one of the leading targets for T-cell recognition of cancer cells. Currently, most studies attempt to identify neoantigens based on predicted affinity to HLA molecules, but the performance of such prediction algorithms is rather poor for rare HLA class I alleles and for HLA class II. Direct identification of neoantigens by mass spectrometry (MS) is becoming feasible; however, it is not yet applicable to most patients and lacks sensitivity. In an attempt to capitalize on existing immunopeptidomics data and extract information that could complement HLA-binding prediction, we first compiled a large HLA class I and class II immunopeptidomics database across dozens of cell types and HLA allotypes and detected hotspots that are subsequences of proteins frequently presented. About 3% of the peptidome was detected in both class I and class II. Based on the gene ontology of their source proteins and the peptide’s length, we propose that their processing may partake by the cellular class II presentation machinery. Our database captures the global nature of the *in vivo* peptidome averaged over many HLA alleles, and therefore, reflects the propensity of peptides to be presented on HLA complexes, which is complementary to the existing neoantigen prediction features such as binding affinity and stability or RNA abundance. We further introduce two immunopeptidomics MS-based features to guide prioritization of neoantigens: the number of peptides matching a protein in our database and the overlap of the predicted wild-type peptide with other peptides in our database. We show as a proof of concept that our immunopeptidomics MS-based features improved neoantigen prioritization by up to 50%. Overall, our work shows that, in addition to providing huge training data to improve the HLA binding prediction, immunopeptidomics also captures other aspects of the natural *in vivo* presentation that significantly improve prediction of clinically relevant neoantigens.

## Introduction

The adaptive immune system has the capacity to elicit anti-cancer CD4^+^ and CD8^+^ T-cell responses, which are triggered by the presentation of cancer-derived antigens as human leukocyte antigen-binding peptides (HLAp) and their recognition by cognate T-cell receptors. HLA class I (HLA-I) and HLA class II (HLA-II) complexes are distinct based on the type of cells that express them, their intracellular processing and loading, and by the type of T-cells that recognize them (1). A dedicated cellular machinery is responsible for the processing of mainly intracellular proteins and their loading on HLA-I complexes, which present these peptides to CD8^+^ T-cells. Similarly, a parallel machinery processes and loads mainly endocytosed extracellular proteins on HLA-II complexes for their presentation to CD4^+^ T-cells. The repertoire of HLA presented peptides (HLAp) is remarkably rich and is collectively called the immunopeptidome (2).

In cancer, HLAp derived from processing and presentation of cancer-specific proteins serve as the leading targets for T-cell recognition. Most antigens identified earlier as cancer-specific have been derived from self proteins. Investigated in hundreds of therapeutic clinical trials, these have been mostly clinically disappointing, partially due to central tolerance mechanisms and the elimination of high-avidity T-cells recognizing such normal proteins (3–6). In recent years, the remarkable clinical efficacy of the immune checkpoint blocking therapies has again motivated researchers to discover the immunogenic T-cell epitopes that mediate disease control or long-term cure (7). The observed correlation between mutational load and clinical efficacy highlights the involvement of mutated neoantigens in tumor rejection, and there is now a growing interest in exploiting such targets in the development of personalized vaccines (8–11).

In recent years, significant technological improvements in genomics along with supportive bio-informatics and *in silico* HLA-binding prediction tools have facilitated major breakthroughs in the discovery of neoantigens encoded by non-synonymous mutations that arise during the process of tumorigenesis and are not expressed by normal cells. Mass spectrometry (MS) technology has confirmed the *in vivo* presentation of neoantigens in murine cell line models (12, 13), human cell lines (14, 15), B-cell lymphomas (16), and melanoma tissues (17). In conjunction, the development of immunological screening techniques has facilitated the detection and isolation of T-cells reactive against such mutated epitopes (18–21). Several studies further showed substantial clinical benefit mediated by the administration of highly enriched populations of neoepitopes-reactive CD4^+^ and CD8^+^ T-cells (22, 23) and neoantigen-based vaccines formulated as RNA (10) or peptides (9). These patients experienced no major toxicity, suggesting that T-cell responses against neoantigens are likely safe.

Currently, the performance of HLA-I ligand interaction prediction algorithms used for identifying potential neoantigens is still rather poor for infrequent HLA-I molecules, for which binding data are limited, and in general for HLA-II molecules (24, 25). Furthermore, predictors of immunogenicity are still immature (26). Inevitably, false positives are included among the predicted neoantigens, which are then included in a vaccine. MS analysis of HLA-I-binding peptides eluted from tissue samples is a promising approach to discover the actual *in vivo* presented immunopeptidome, including the neoantigens (17). The more specific targeted MS analysis may be used to further validate presentation of *in silico* predicted neoantigens (12). With the current MS instrumentation, MS-based immunopeptidomics approaches have limited sensitivity and are only applicable to a small fraction of patients due to the large amount of biological sample that is required (typically 1 cm^3^ of tissue or 1 × 10^8^ cells in culture). Furthermore, they are currently performed in only a few professional labs due to the complexity of these experiments (27).

In addition, interrogating the properties of the thousands of different source-proteins of HLA ligands has identified additional biological determinants, such as their level of translation and expression, turnover rate, proteasomal cleavage specificities, length, and biological functions. Integrating such variables into a single predictor further improves the accuracy of prediction (28, 29). Specifically, recent MS immunopeptidomics studies suggested that HLA-I ligands are not randomly distributed along the proteins’ sequences but are located within “hotspots” (17, 28), which fit proteasomal cleavage, peptide processing and HLA-binding rules.

In recent years, it has become common practice in proteomics research to submit MS/MS data to repositories in order to make them available for further research (30). More recently, this practice is also being followed in the field of immunopeptidomics (17, 28, 31). So far, the large body of publically available MS/MS data has been used for training of HLA-I binding prediction (29, 32–34) or to build spectral libraries (35). Although MS-based immunopeptidomics analysis can be directly applied today only to a small number of patients, its emerging use can reveal crucial information on the rules underlying the biogenesis of the immunopeptidome. Indeed, while hunting for neoantigens, such immunopeptidomics MS studies produce massive amount of highly valuable ligandomic data that can be used to refine known HLA-I-binding motifs and to reveal HLA-I-binding specificities of yet unexplored alleles (32, 33). Here, we propose another way to valorize available immunopeptidomics MS/MS data.

We first computationally overlaid HLA-II peptidomics data on top of HLA-I data to highlight the subpopulations of cellular proteins that are naturally accessible and presented by each of the HLA-I and HLA-II presentation machineries and those presented by both (HLA-I/II). Based on the functional annotation of the source proteins and the peptide’s length we propose that the HLA-I/II peptides may be processed by the cellular class II presentation machinery within the endosome-lysosome compartments, in a proteasome-independent cross-presentation pathway. Since priming both CD8^+^ and CD4^+^ T cell responses would lead to optimal and long lasting immune response required for elimination of tumors *in vivo*, these cross-presented peptides are of particular importance. Next, we provide evidence that data-driven prioritization of predicted neoantigens based on observed “hotspots,” which are subsequences of proteins frequently detected in MS/MS immunopeptidomic datasets, will enrich the list of proposed targets with the most likely presented neoantigens. These hotspots reflect the propensity of protein subsequences to produce HLA peptides averaged over many allotypes and provide complementary information to classical HLA-binding prediction. We show as a proof of concept that by including MS-based hotspot scores into the prioritization scheme we are able to improve the prediction by up to 50%. We envision that as MS-based HLA-I and HLA-II immunopeptidomics datasets become more exhaustive, “hotspot” driven prioritization will have a substantial impact on the selection of neoantigens for vaccination.

## Material and Methods

### Cell Lines, Tissues, and Antibodies

Detailed information about the biological samples that were included in this database is provided in Table S1 in Supplementary Material. Informed consent of the participants was obtained following requirements of the institutional review board [Ethics Commission, University Hospital of Lausanne (CHUV)]. W6/32 (anti-pan-HLA-I) and IVA12 (anti-pan-HLA-II) monoclonal antibodies were purified from the supernatant of HB95 and HB145 cells, respectively, as previously described (17). We cross-linked the antibodies to Protein-A Sepharose beads (Invitrogen, CA, USA) with 20 mM dimethyl pimelimidate in 0.2 M sodium borate buffer pH9.

### Purification of HLA-I Complexes

We included here also unpublished immunopeptidomics data of HLA-I and HLA-II peptides extracted from several biological replicates per cell line or patient material. The cell counts ranged from 1 × 10^8^ to 5 × 10^8^ cells or up to 2 g of tissue per replicate. Purification from these additional samples was performed as previously described (17, 33). Shortly, snap-frozen tissue samples were homogenized for 10 s on ice using ULTRA-TURRAX (IKA, Staufen, Germany) in a tube containing 5–10 ml of lysis buffer and incubated at 4°C for 1 h. Cell pellets were resuspended in 5 ml lysis buffer and incubated similarly. Lysis buffer contained 0.25% sodium deoxycholate (Sigma-Aldrich), 0.2 mM iodoacetamide (Sigma-Aldrich), 1 mM EDTA, 1:200 Protease Inhibitors Cocktail (Sigma, MO, USA), 1 mM Phenylmethylsulfonylfluoride (Roche, Mannheim, Germany), 1% octyl-beta-D glucopyranoside (Sigma). The lysates were cleared by centrifugation with a table-top centrifuge (Eppendorf Centrifuge 5430R, Schönenbuch, Switzerland) at 4°C at 14,200 rpm for 20 min. Immuno-affinity purification from tissues was performed by passing the cleared lysates through Protein-A Sepharose beads, then through Protein-A Sepharose beads covalently bound to W6-32 antibodies, and finally through beads covalently bound to IVA12 antibodies. Purification from cell line lysates required only the two last affinity columns. Affinity columns were then washed with at least 6 column volumes of 150 mM NaCl and 20 mM Tris–HCl (buffer A), 6 column volumes of 400 mM NaCl and 20 mM Tris–HCl and lastly with another 6 column washes of buffer A. Finally, affinity columns were washed with at least 2 column volumes of 20 mM Tris HCl, pH8. HLA complexes were eluted by addition of 1% trifluoroacetic acid (TFA, Merck, Darmstadt, Switzerland) for each sample. To further purify the peptides, the elution samples were loaded separately on Sep-Pak tC18 (Waters, MA, USA) cartridges, which were pre-washed with 80% acetonitrile (ACN, Merck) in 0.1% TFA and 0.1% TFA only. After loading, cartridges were washed twice with 0.1% TFA before separation peptides were eluted with 30% ACN in 0.1% TFA. The peptide samples were dried using vacuum centrifugation (Eppendorf Concentrator Plus, Schönenbuch, Switzerland) and re-suspended in a final volume of 12 μL 0.1% TFA. For MS analysis, we injected 5 μL of these peptides per run.

### LC–MS/MS Analysis of HLA-I Peptides

Measurements of HLA-I and HLA-II peptidomics samples were acquired using the nanoflow UHPLC Easy nLC 1200 (Thermo Fisher Scientific, Germering, Germany) coupled online to a Q Exactive or Q Exactive HF Orbitrap mass spectrometers (Thermo Fischer Scientific, Bremen, Germany) with a nanoelectrospray ion source as previously described (33). We packed an uncoated PicoTip with diameter of 50 cm × 75 µm and 8 µm tip opening with a ReproSil-Pur C18 1.9 µm particles and 120 Å pore size resin (Dr. Maisch GmbH, Ammerbuch-Entringen, Germany) resuspended in Methanol. The analytical column was heated to 50°C using a column oven. Peptides were eluted with a linear gradient of 2–30% buffer B (80% ACN and 0.1% formic acid) at a flow rate of 250 nL/min over 90 min.

Data were acquired with data-dependent “top10” method, which isolates the ten most intense ions and fragments them by higher-energy collisional dissociation with a normalized collision energy of 27%. The MS scan range was set to 300–1,650 *m*/*z* with a 60,000 (200 *m*/*z*) resolution and a target value of 3e6 ions. The ten most intense ions were sequentially isolated and accumulated to an AGC target value of 1e5 with a maximum injection time of 120 ms and MS/MS resolution was 15,000 (200 *m/z*). The peptide match option was disabled. Dynamic exclusion was set for 20 s.

### Data Analysis of HLA Peptides

We employed the MaxQuant computational proteomics platform (36) version 1.5.3.2 to search the peak lists against the UniProt database (Human 85,919 entries, May 2014) and a file containing 247 frequently observed contaminants. All MS/MS datasets were processed in one batch using a global spectrum level false discovery rate (37) cutoff of 1%. Protein N-terminal acetylation (42.010565 Da) and methionine oxidation (15.994915 Da) were set as variable modifications. The second peptide identification option in Andromeda was enabled. The enzyme specificity was set as unspecific. The initial allowed mass deviation of the precursor ion was set to 6 ppm and the maximum fragment mass deviation was set to 20 ppm.

### Compiling the Immunopeptidomics Database

An in-house Java program^1^ based on the MzJava class library (38) was used to parse the MaxQuant results and organize them in a database (*ipMSDB*). The matching and scoring between *ipMSDB* and query peptides was done by another in-house Java program and all further data analysis and visualization was performed in R^2^ if not otherwise indicated.

### Gene Ontology (GO) Enrichment Analysis and Tree-Map Visualization

The GO enrichment analysis of the source proteins of the presented HLA peptides was performed on the Panther webpage^3^ (39). All human proteins were taken as a background and compared to the different protein lists based on the biological process classification. Proteins were quantified by their type I, II, or I/II peptide counts, respectively. A statistical overrepresentation test was performed and the resulting *p*-values were corrected for multiple testing and a *p*-value threshold of 0.05 was applied.

For visualizing of the protein lists, we used the Proteomaps tool^4^ (40), which is based on the KEGG protein annotation. We used the same protein lists and the same protein peptide counts as for the GO analysis. The resulting Veronoi-tree-map images were slightly edited for better visibility of the text.

### HLA-I, HLA-II, and HLA-I/II Density Profiles and Correlation between Them

HLA-I, HLA-II, and HLA-I/II density profiles were calculated from *ipMSDB* by summing up for each amino acid in a protein sequence the number of HLA-I, HLA-II, and HLA-I/II peptides covering this amino acid. In order to display profiles of HLA-I peptides of typical length, HLA-I peptides shorter than 15 amino acids were also considered separately. The correlation between profiles was calculated in a way that reflects the overlap of the main peaks in a profile ignoring smaller peaks.

Profiles for NetMHCpan version 3.0 (25) HLA-I-binding affinity prediction for the GILT, SEM4D, and MITF proteins were obtained from the web page.^5^ All HLA supertype representatives and peptides of length 9–11 were selected. All predicted strongly or weakly binding peptides were retained. The profiles are the NetMHCpan scores of the representative binders summed up over all strong or weak binding peptides for each amino acids of the protein.

### Training Predictor and Cross Validation

In order to test whether our *ipMSDB* based features are able to improve the prioritization of predicted immunogenic peptides, we used data from a recent publication by Stronen et al. (41): 1,034 HLA-I peptides of length 9–11 carrying non-synonymous somatic mutations were obtained by genome sequencing from 3 melanoma patients and were screened with T-cell assays for immune recognition. 16 out of 1,034 neoantigens turned out to be immunogenic. Stronen et al. calculated several features for both mutated and wild-type (wt) peptides to enable prioritization of neoantigens: best predicted binding affinity to one of the patients HLA-I alleles (*mutAffinity, wtAffinity*), predicted HLA-I-peptide complex stability (*mutPeptideStability, wtPeptideStability*), proteasomal cleavage probability (*mutCleavProb, wtCleavProb*), number of mutated and wt reads (*mutReads, wtReads*), and RNA abundance (*rnaExpr*). Here, we added three MS-based prediction scores, which evaluate how well the wt counterpart of the predicted mutated peptide is represented in *ipMSDB*. The first score (*nrMatchingPeptides_I*) operates on the protein level and counts the number of all wt HLA-I peptides per protein in *ipMSDB*. The other two scores (*matchScore_I* and *exactMatchScore_I*) operate on the peptide level. In order to calculate *matchScore_I* for a peptide, we sum up the HLA-I density profile height over the position of the peptide. If the peptide is found on multiple proteins and/or several times on the same protein in *ipMSDB*, we take the highest of all the *matchScore_I* values*. exactMatchScore_I* is equal to *matchScore_I* if there is an exact wt peptide match in *ipMSDB* and 0 otherwise.

In order to compare our *ipMSDB* predictors to the features described in Stronen et al. individually, all the 1,018 control and 16 immunogenic peptides were used. We applied a support vector machine (SVM) regression (42) with Gaussian kernel to compare the predictive power of feature sets. The R package e1071, which is an interface to the LIBSVM SVM implementation (43), was used for this purpose. Other than kernel selection no optimization was performed and all SVM parameters were kept at their default values. Combining several features means that each peptide is represented by a feature vector in a *N*-dimensional space, where *N* is the number of features. The task of the SVM regressor is to grade this feature space with values between −1 and 1, where values close to one represent “immunogenic” regions of the feature space. To calculate this peptide immunogenicity grading, the SVM needs to learn from training data, and in order to evaluate the quality of this learning, the grading is compared to independent test data. If the immunogenic test peptides lie in regions in the feature space with grade close to 1 and the control test peptides in regions with grade close to −1, the learning has worked well. To perform the learning and independent testing, the control and immunogenic peptide lists were both randomly split into equally sized training and test parts. One part of the control and one part of the immunogenic peptide list go to the training set (half of all peptides), the other parts to the test set (the other half of all peptides). The SVM was trained on the training set and the trained SVM regression was used to rank the peptides in the test set. The number of immunogenic peptides in the 20 top ranked test peptides was calculated as the prediction performance (value between 0 and 8). The process was repeated 2,000 times in order to calculate average performance values and their standard deviations.

## Results and Discussion

### Assembling Large-scale Human Immunopeptidomics Database

**Figure 1:**
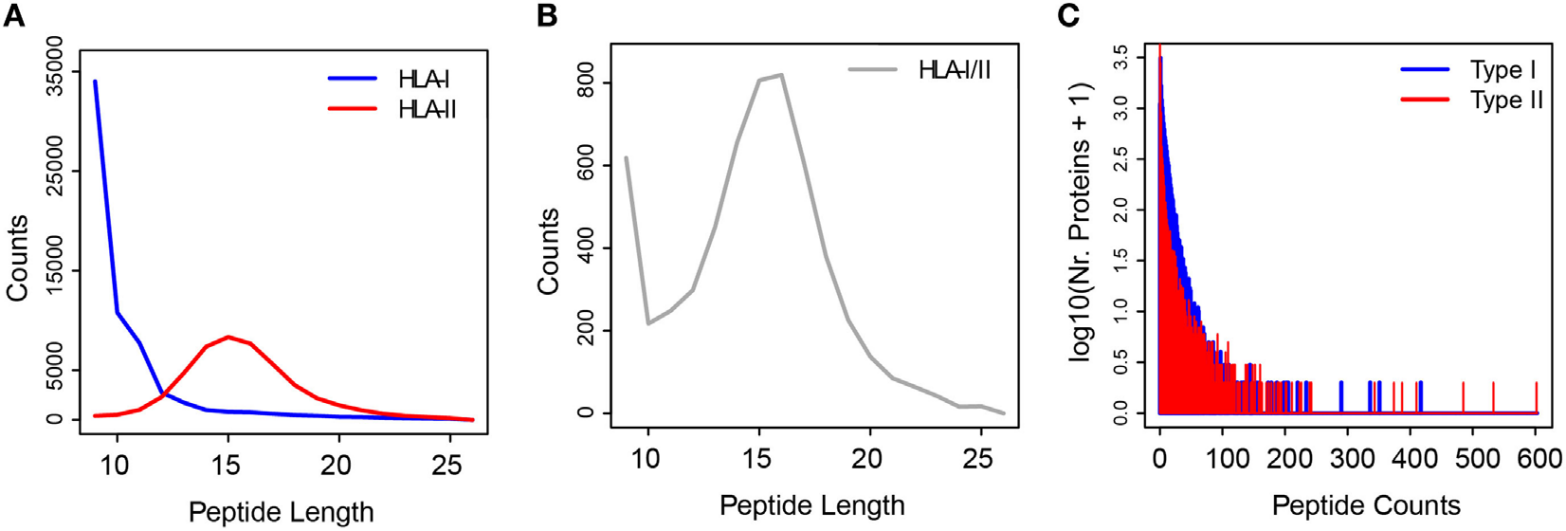
**(A)** Length distributions of HLA-I (in blue) and HLA-II (in red) peptides. **(B)** Length distribution of HLA-I/II peptides samples. **(C)** Histogram of the number of peptides per “type I” (blue) and “type II” (red) source proteins.

The experimental extraction procedure of HLA peptides highly enriches for the true HLA ligands. More than 95% of the HLA-I peptides we identified by MS matched the typical properties of sequence length and binding motifs that are necessary for binding to the different HLA-I allotypes (31). In order to build the *ipMSDB* database of HLA peptides, we compiled data from our recent published immunopeptidomics experiments (17, 31, 33, 44) and we added unpublished data (Table S1 in Supplementary Material). Altogether, *ipMSDB* represents an in-depth repertoire of HLA-I and HLA-II peptides purified separately from dozens of different human cell lines and tissues covering many HLA allotypes. Currently, our *ipMSDB* includes 15,422 protein groups with at least one valid peptide match (only MaxQuant leading razor proteins were considered) identified from 67 different biological samples, mainly B-cells (13 samples), T-cells (4 samples), and melanoma tissues (35 samples). At the peptide level, this corresponds to 131,402 unique peptides detected in HLA-I peptide samples and 66,420 unique peptides detected in HLA-II peptide samples. The length distribution (mostly 9 to 11 -mer peptides) of the identified HLA-I peptides highlights the purity of the peptidome (Figure 1A). Unlike HLA-I complexes, HLA-II complexes presented families of longer peptides (mainly 13–17 amino acids) (Figure 1A), sharing the core binding region of typically 9 amino acids. The binding restrictions of HLA-II peptides are still rather poorly understood and technically it is more challenging to retrieve them directly from immunopeptidomics data as a way to estimate the purity of HLA-II peptidome samples. We expect that the HLA-II peptidomes have similar high purity level because we purified them similarly to the HLA-I. Also, when cells lack HLA-II expression no peptides are detected (17). Interestingly, 6,819 unique HLA-I/II peptide sequences (3.4%) in *ipMSDB* were detected in both HLA-I and HLA-II samples. Figure 1B reveals that their length distribution is a mixture of the class I and class II modes.

Remarkably, the broad distribution of the number of peptides presented as HLA-I and HLA-II peptides per source protein implies that the proteins are not randomly selected for presentation (Figure 1C; Figure S1A in Supplementary Material). As we have shown previously (31), the number of HLA-I peptides per protein depends on the protein length (Figure S1B in Supplementary Material), but the HLA presentation of proteins also depends on many other factors. The assembly of the above database has allowed preliminary observations that provide important hints on the biogenesis of the immunopeptidome. These could be exploited in the development of algorithms for optimizing the prediction of neoantigens. Our main working hypotheses are presented below.

### Hint 1: The Proteome Is Selectively Sampled for Antigen Presentation

**Figure 2:**
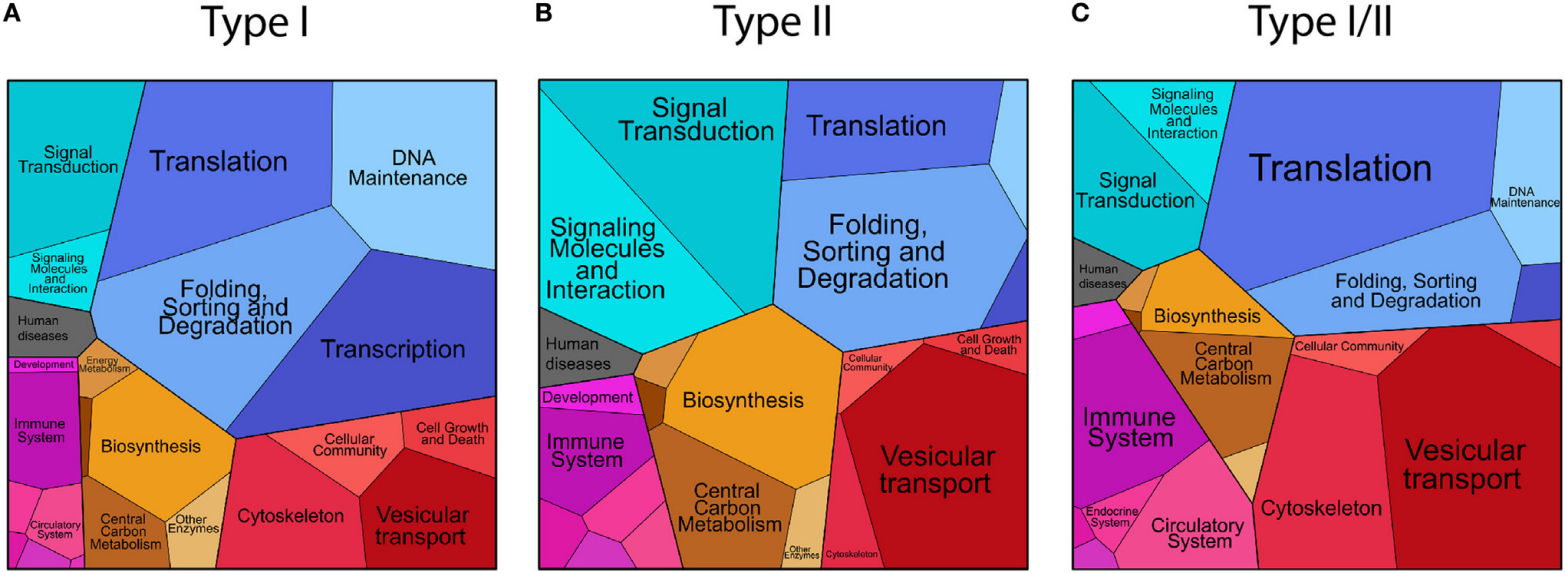
Proteomaps visualization of the level of presentation (number of unique peptide sequences) of the source proteins of “type I” **(A)**, “type II” **(B)**, and “type I/II” **(C)** groups classified according to their annotated cellular functions.

We compared the characteristics of the source proteins presented as HLA-I and HLA-II peptides in terms of the biological process GO annotations (see Materials and Methods for details). We further visualized the data with the Proteomaps tool (see Materials and Methods), which is based on the KEGG protein annotation. The Proteomaps tree-map visualization tool shows quantitative composition of proteomes arranged in multiple levels. As a pseudo quantitative score we used the number of assigned HLA peptides per protein in *ipMSDB*. On the lowest level, each protein is represented by a polygon, whose area reflects the number of HLA-I, HLA-II or HLA-I/II peptides, times the protein length, respectively. Functionally related proteins according to a KEGG hierarchy tree were arranged in adjacent and similarly colored regions. On higher levels, similar proteins were grouped into regions. We investigated cellular proteins separately for the following groups:

1. Cellular proteins presented as HLA-I peptides, collectively named “type I.” A large fraction comprising 93.2% of the source proteins in *ipMSDB* were presented as HLA-I peptides. HLA-I molecules present most of the cellular proteome. GO annotation enrichment analysis (Table S2 in Supplementary Material) revealed that compared to the reference human proteome, “type I” were enriched in the following biological processes: nuclear chromosomes (*p-*value = 2.93E−02), nucleus (7.02E−10), and nuclear envelope (2.10E−03) and also lysosome (1.01E−02), endosome (4.67E−04) the Golgi apparatus (4.42E−02), vacuole (2.69E−04) and more general annotation like the ribosome (3.54E−23) and the cytoskeleton (6.81E−07). The MHC protein complex was enriched (6.88E−03), while membrane proteins in general were depleted (3.87E−02). The proteomaps were in agreement with the GO annotations enrichment analysis (Figure 2A and in more details in Figure S2 in Supplementary Material). This pattern was independently observed in B-cells, T-cells and in melanoma tissues. Differences were partially related to differences in protein expression between cell lineages and/or between *in vivo* tissues and cells growing in culture. For example, ribosomal and cytoskeleton proteins were more prominent in melanoma tissues than in B- and T-cells, while proteins related to DNA replication were presented more in rapidly dividing cells growing in culture (Figure S3A in Supplementary Material).
2. Cellular proteins presented as HLA-II peptides were collectively named “type II.” “Type II” proteins were enriched in the lysosome (*p-*value = 7.05E−04), endosome (1.22E−06) Golgi apparatus (1.41E−05), vacuole (2.72E−06), ribosome (6.08E−32), and the cytoskeleton (2.55E−03) biological processes (Table S2 in Supplementary Material). The MHC protein complex was also similarly enriched (3.31E−04) as it was in “type I,” while the SNARE complex (1.37E−02) and the vesicle coat (3.37E−02), the proton-transporting ATP synthase complex located to the mitochondria (2.3E−02) were uniquely enriched in “type II” (Figure 2B; Figure S4 in Supplementary Material). Some proteins differed in their presentation as HLA-II peptides in melanoma tissues compared to cells growing in culture. For example, complement and coagulation cascade proteins and hemoglobin were detected only in the tissues (Figure S3A in Supplementary Material).
3. Cellular proteins presented by both machineries, in which at least one HLA-I/II peptide sequence was detected are called “type I/II” proteins. This group of proteins was similar to the “type II,” as they were enriched in the lysosome (*p-*value = 9.86E−05), Golgi apparatus (6.79E−03), vacuole (7.59E−05), vesicle coat (1.61E−02), ribosome (4.38E−45), and MHC protein complex (3.76E−06) biological processes, while uniquely to the “type I/II” the extracellular space was enriched (5.57E−05) (Table S2 in Supplementary Material). The typical chromosome or nucleus related proteins that are characteristic for “type I” were not significantly enriched here. Compared to “type I” proteins, “type I/II” proteins comprised of less DNA association, and similarly to the “type II,” they included more proteins related to vesicular transport (Figure 2C; Figures S3A and S5 in Supplementary Material).

Collectively, these results indicate that the sampling of the self proteome for presentation on HLA-I and on HLA-II complexes is not random and the cellular localization of proteins, possibly also related to the mechanism of their degradation, has an impact. More than that, a subset of the proteome is presented by both machineries and resembles “type II” source proteins.

### Hint 2: HLA-I/II Peptides Suggest a Cross-Talk between HLA-I and HLA-II Presentation Pathways

3.4% of peptide sequences in *ipMSDB* were detected as HLA-I/II peptides. Such long peptides detected in HLA-I peptidome could also be a technical artifact of contaminating HLA-II peptides that occurs during the purification. However, several main observations argue against this option: first, a significant part of the long HLA-I peptides fit the P2/PΩ-anchor mode of binding to the expressed HLA-I allotypes. We showed this for the UWB289 ovarian cancer cells that do not express HLA-II, and melanoma tissues from Mel15 and Mel16 patients form which both HLA-I and HLA-II peptidomes were obtained (Figure S6 in Supplementary Material). The remaining peptides could still bind with alternative internal anchors leaving the ends of the peptides to protrude beyond the binding groove (45, 46). Second, we calculated the proportion of long peptides (equal or longer than 14-mers) detected in cell lines or tissue samples that express HLA-II, and those that lack HLA-II expression, i.e., in which no or only sparse amounts of less than 100 HLA-II peptides could be detected by MS. The average proportion of longer HLA-I peptides in the group expressing HLA-II peptides was 5.6%, whereas the average proportion in the group lacking HLA-II peptides was 4.8% which was not significantly different (standard deviation is 3%). Therefore, it is very unlikely that the long HLA-I peptides were contaminations from HLA-II peptides. Furthermore, HLA-II peptides were purified from the lysates after the HLA-I had been purified, which minimized the chance that HLA-II peptides would contaminate class I peptidome samples. No HLA-II peptides could be detected in samples from which thousands of HLA-I were identified, which supports the claim that there is no significant cross contamination related to sample handling. We further elaborate on the possible biogenesis of the HLA-I/II peptides below.

**Figure 3:**
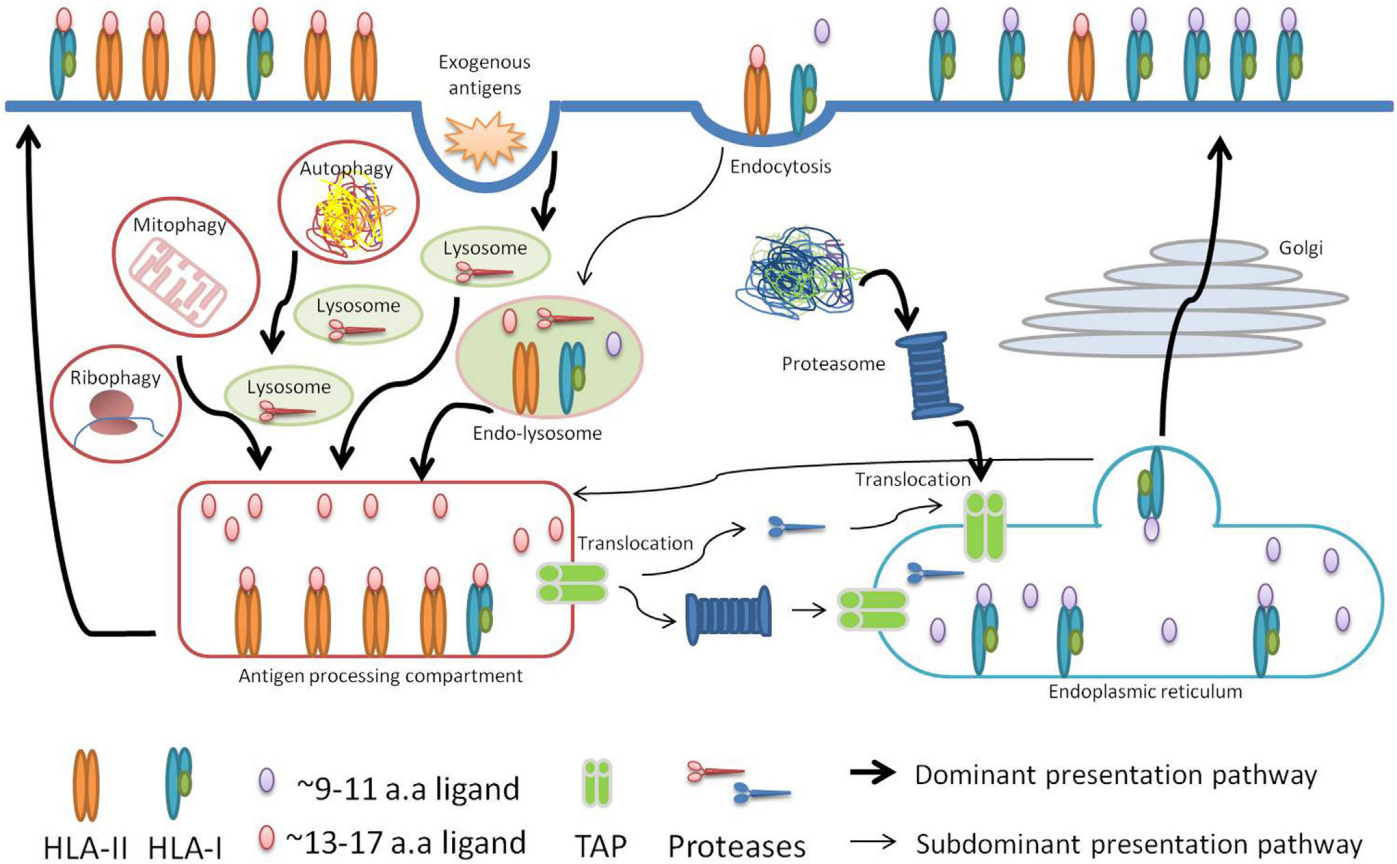
Schematic visualization of our hypothesis. In cancer or upon infection, professional antigen-presenting cells take up antigens released by dying cells, degrade them in the endosome-lysosome compartments, and present their longer peptides as either HLA-II or HLA-I peptides in a proteasome-independent manner. In addition, shorter HLA-I peptides are presented *via* the conventional proteasome-dependent class I pathway. In case cells are directly infected with intracellular pathogens or at steady state conditions, autophagy may similarly lead to the presentation of longer peptides, from the pathogens or from the self proteome, including as HLA-I.

Cross-presentation has been investigated for many years and it has been shown to be central for the priming of naïve T-cells against exogenous antigens. These antigens are taken up by professional antigen-presenting cells that process and consequently present them on HLA-I molecules (47). Cross-presentation happens *via* two orthogonal routes: a proteasome- and TAP-independent route where proteins digested in endosomes are loaded on HLA-I molecules imported into the endosomes, and a proteasome- and TAP-dependent route where endosomal proteins are exported to the cytosol and processed by the HLA class I presentation machinery (Figure 3).

Several lines of evidence, which we discuss below, led us to propose that “type I/II” source proteins are processed partially by the machinery involved in cross-presentation in the endosome-lysosome compartments. We hypothesize that cross-presentation of peptides cleaved in the endosomes consequently leads to the generation and loading of longer HLA-I peptides that are likely to be in common with the peptides generated by the class II processing machinery, and stem from the same source proteins (Figure 3). It is important to note that cross-presented peptides may also be generated after the polypeptides have been transferred from the lysosome–endosome compartments into the cytosol. Following the conventional class I presentation pathway that is proteasome- and TAP-dependent, these peptides will then become indistinguishable from the normal pool of HLA-I peptides characterized with a typical length (9–11 aa) (47).

We observed that HLA-I/II peptides stemmed mainly from self proteins localized within the endosome-lysosome compartments and were enriched with phagosomal structural proteins and phagosomal cargo proteins that are degraded by autophagy (Figure 2). Among them are ribosomal proteins and mitochondrial proteins that may be selectively degraded and eliminated in the processes called ribophagy (48) and mitophagy (49), respectively. Previously, we studied HLA-I presentation in several cell lines and we have shown that ribosomes and mitochondrial proteins are presented to a higher extent than what would be expected from their abundance (31). One example of a protein that belongs to the “type I/II” group is the “probable serine carboxypeptidase” (CPVL) localized in phagosomes. It may be involved in the digestion of phagocytosed particles in the lysosome and in trimming of peptides for antigen presentation (50). Another example is the PMEL protein from which several peptides were detected to be presented on HLA-I and HLA-II complexes. PMEL is involved in melanosome formation and disintegration of melanosomes is assumed to take place in the lysosomes (51). Furthermore, the autophagic pathway has a substantial role in the degradation of melanosomes in keratinocytes (52). The confined space within the endosome-lysosome compartments may indeed favor cross-presentation of this set of proteins and could also explain how low abundant proteins may still be presented with multiple ligands and out-compete very abundant proteins. Furthermore, HLA-I/II peptides seem to be more prominent in B-cells and T-cells compared to melanoma tissues where class II presentation machinery might not be fully functional (Figure S3B in Supplementary Material).

Based on these observations, we hypothesize that in cancer or upon infection, professional antigen presenting cells that take up antigens released by dying cells and degrade them in the endosome-lysosome compartments, would present their longer peptides as either HLA-II or HLA-I peptides generated through the proteasome-independent pathway (Figure 3). Furthermore, in case cells are directly infected with intracellular pathogens or at a steady state condition, autophagy may lead to the presentation of longer HLA-I peptides from the pathogens or from the self-proteome. For example, a recent study investigated the HLA-I peptidome of cells upon infection with the intracellular pathogen *Toxoplasma gondii* (45), and reported that the *T. gondii* ligands were significantly longer than host ligands. The average length of *T. gondii* ligands was 14.6 amino acids compared to 11.4 amino acids of host ligands for infected and 9.8 amino acids for uninfected cells. Furthermore, they observed that the long ligands did not follow the P2/PΩ-anchor binding mode of HLA-I but instead were predicted to bind *via* a canonical N-terminal binding core preceding the C-terminal extension. Both the length preference and the mode of binding of these peptides may be explained by the alternative processing we describe here. Notably, the 9–11-mers could potentially be mainly driven by ER-resident chaperones and peptidases that are known to play a role in the ER conventional class I presentation pathway.

### Hint 3: Protein Hotspots Are Selectively Sampled for Antigen Presentation

**Figure 4:**
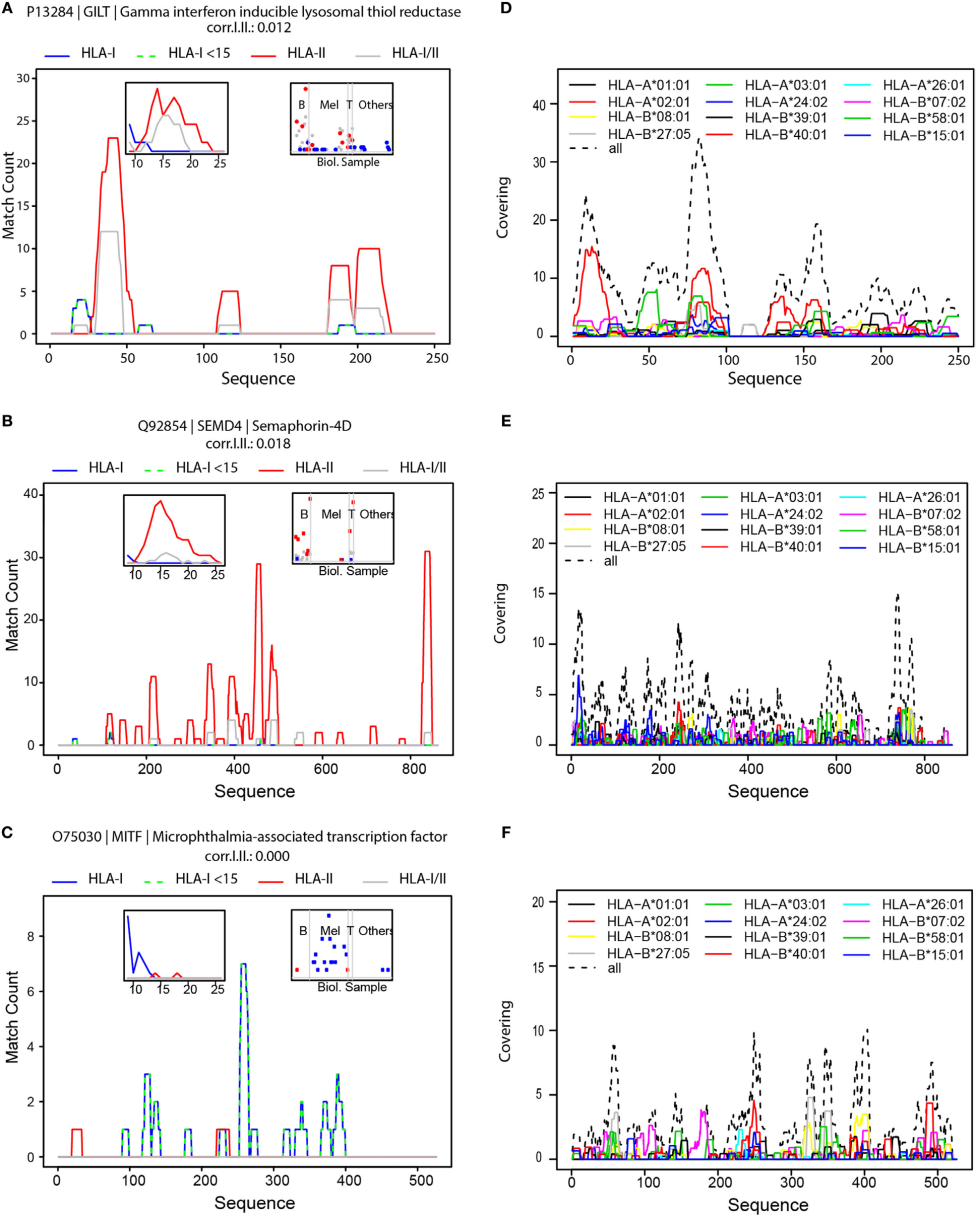
Hotspots of mass spectrometry-detected HLA-I and HLA-II binding peptides and the landscape of predicted HLA-I peptides. Visualization of hotspot of HLA peptides from GILT **(A)**, SEMD4 **(B)** and MITF **(C)**; density profiles of HLA-I peptides (in blue), HLA-I peptides shorter than 15-mers (in green dashed line), HLA-II peptides (in red), and HLA-I/II peptides (in gray). The left inserts are histograms of the length of HLA-I, HLA-II and HLA-I/II peptides, and the right inserts visualize the number of peptides detected in the different samples, grouped as B-cells, Melanoma, T-cells and other samples. Visualization of the landscape of possible presentation of GILT **(D)** SEMD4 **(E)** and MITF **(F)** using alignment of peptide sequences predicted by NetMHCpan to bind any of the 12 HLA-I supertypes.

Interestingly, we have noticed that there are “hotspots” of antigen presentation within proteins, and that domains within proteins are presented at a higher extent. We separately aligned HLA-I, HLA-II, and HLA-I/II peptides to the protein sequences. As an example, we show the hotspots we detected for the gamma-interferon-inducible lysosomal thiol reductase (GILT) protein (UniProt P13284), the semaphorin-4D (SEM4D) protein (Uniprot Q92854), and the microphthalmia-associated transcription factor (MITF, Uniprot O75030) (Figures 4A–C, respectively). More examples are provided in Figures S7A–F in Supplementary Material. GILT is the only enzyme known to catalyze disulfide bond reduction in the endocytic pathway. It facilitates presentation of a subset of HLA peptides from disulfide bond-containing antigens (53). GILT is expressed constitutively in antigen-presenting cells and is induced by gamma-interferon in other cell types. It has an important role in HLA-II-restricted antigen processing and was reported to be expressed in most of primary and metastatic melanomas (54). Indeed, we also detected GILT in B- and T-cells and in melanoma tissues (Figure 4A, right inset). SEM4D belongs to the semaphorin family and it regulates the sensitivity of the B-cell antigen receptor that is required for proper B-cell homeostasis (55). We observed that SEM4D was mainly presented in B-cells and also in T-cells (Figure 4B). The length distribution of peptides derived from GILT and SEM4D (left insets in Figures 4A,B) reveals that the longer HLA-I peptides were also detected as HLA-II peptides. Therefore, these peptides were mainly HLA-I/II (gray line). Globally, such HLA-I/II hotspots overlapped significantly more often with HLA-II hotspots than with HLA-I hotspots (Figure S8 in Supplementary Material).

Naturally, tissue specificity will further restrict the presentation of the antigens. For example, MITF is a transcription factor that regulates the expression of genes with essential roles in cell differentiation, proliferation and survival (56). MITF plays an important role in melanocyte development by regulating the expression of tyrosinase (TYR) and tyrosinase-related protein 1 (TYRP1) (56). Indeed, we also identified MITF, TYR, and melanocyte protein PMEL ligands almost exclusively in the melanoma tissues (Figure 4C; Figures S7D,E in Supplementary Material).

It is important to note that HLA-binding affinities cannot accurately predict the hotspots we detected for these proteins (Figures 4D–F). Therefore, the immunopeptidomics data provide critical additional information to capture the true *in vivo* presented ligandome. The height of the hotspots and the distribution of hotspots along the protein sequence reflect the level of its presentation. Hotspots may be related to sequence and structure dependent proteasomal or endosomal cleavage preferences. Alternatively, hotspots may be merely the outcome of *de facto* presentation of the rather more stable polypeptides surviving the highly proteolytic cytosolic environment as the expected half life of peptides in the cytosol of living cells is 6–10 s (57). Furthermore, some posttranslational modifications may interfere with protein cleavage and with binding of the modified peptides to the HLA, eliminating them from the presented repertoire. Currently, no prediction algorithms incorporate these factors. Since our database comprises of peptidomes of dozens of different HLA allotypes and binding specificities, these hotspots reflect an average propensity of a protein sub-sequence to be presented on a HLA molecule. Hotspots may encompass HLA peptides with N′ or C′ terminal extensions of several amino acids to accommodate peptides that fit a variety of HLA-binding specificities. Hotspots of “type I” proteins are highly enriched in 9–11 HLA-I peptides (Figures S7A,B in Supplementary Material). Similarly, hotspots of “type II” proteins are longer, and the length distribution of their peptides is centered on 15-mer peptides (Figure S7C in Supplementary Material).

### Hints on Immunopeptidomics Biogenesis Can Be Applied for Prioritization of Neoantigens

MS-based immunopeptidomics is a powerful approach to shed light on the selective sampling of the proteome. Because it captures the actual presented peptidome, it may bypass the need to computationally predict ligands. Therefore, it is also a promising method to directly identify presented neoantigens. However, enough tumor tissue is available only for the minority of patients and only rarely such neoantigens can be detected due to current limitation in sensitivity. Therefore, the widespread approach for identification of neoantigens for personalized cancer vaccines is still based on *in silico* predictions of the binding affinity to the respective HLA class I allotypes of 9–11-mer peptide sequences harboring the non-synonymous mutations. RNA expression data are further interrogated to exclude non-expressed genes. This *in silico* approach has high false positive rate and, consequently, the list of proposed targets must be further filtered to enrich for true positives. In addition, as we have just discussed above, some HLA ligands are longer and do not conform to the classical binding mode, hence are expected to be false negatives. All the hints presented above could be incorporated into predictors for neoantigen immunogenicity. Here, we focus on Hints 1 and 3.

**Figure 5:**
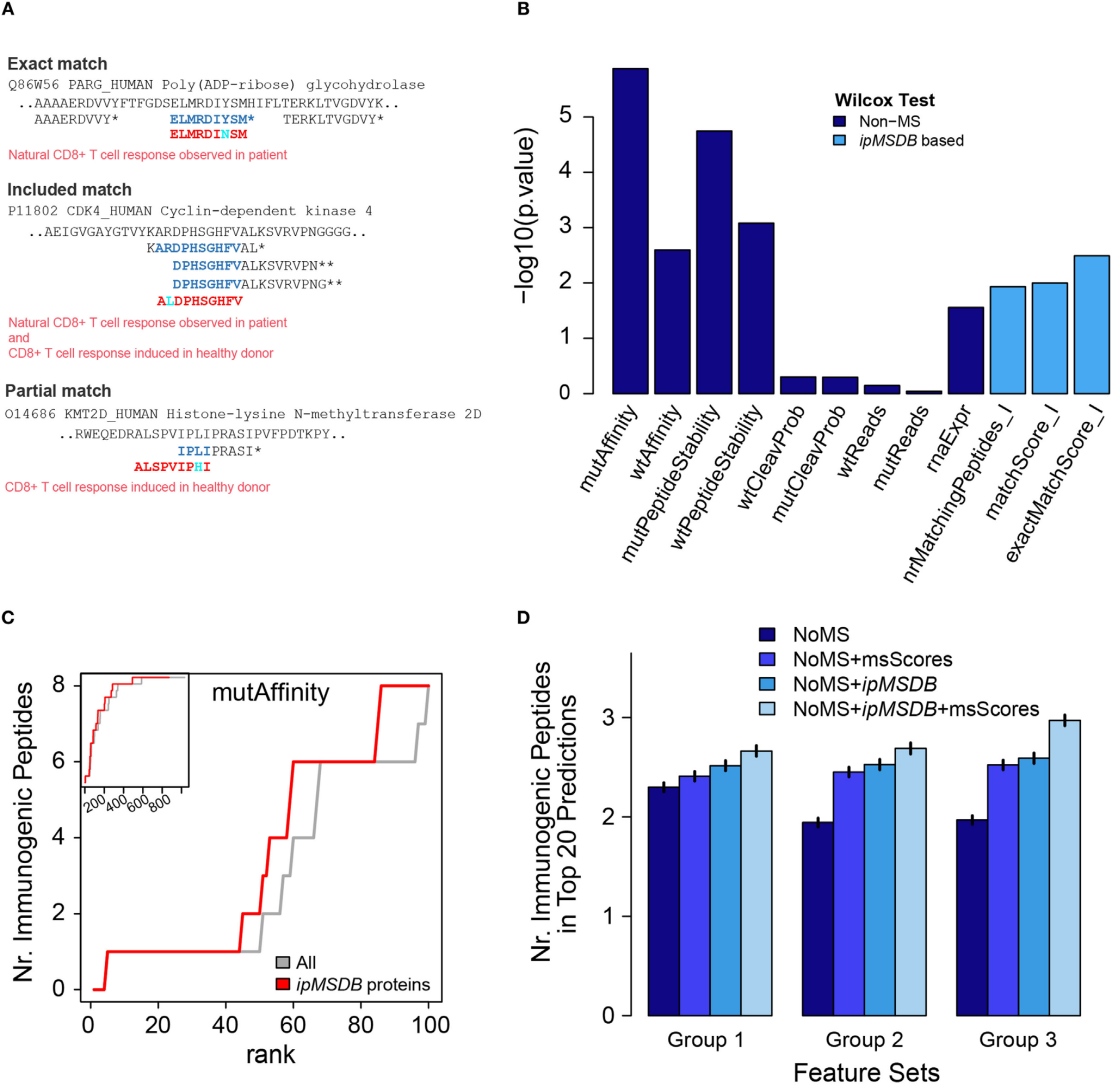
**(A)** Examples of matches of confirmed immunogenic neoantigens from Stronen et al., with peptides in *ipMSDB*. Predicted neoantigen sequences are in red, overlapping amino acids within peptides detected by mass spectrometry (MS) are in blue and mutation are in cyan. Matches can be “exact,” signifying that the exact wild-type (wt) counterpart of the neoantigen sequence was detected by MS, “included,” i.e., the neoantigen is included within the sequence of a longer wt peptide detected by MS, or “partial,” i.e., the neoantigen is partially overlapping at the position of the mutation with the wt counterpart detected by MS. *HLA-I peptides, **HLA-II peptides. **(B)** The higher the −log_10_ of Wilcox-test *p*-values, (i.e., the smaller the pValue), the more different the distribution of immunogenic peptide values is to the distribution of non-immunogenic peptide values. See Ref. (41) for details about the non-MD scores. **(C)** Predicted peptides were ranked by *mutAffinity* score (HLA-binding affinity of mutated peptide) and the number of correctly predicted immunogenic peptides (maximally 16) is plotted against the rank. Gray line represents before and red line after removal of proteins not present in *ipMSDB*. The main figure shows the first 100 ranks, whereas the inset shows all 1,034 ranks with the *y*-axis ranging from 0 to 16. **(D)** Average number of correctly predicted immunogenic peptides in the top 20 peptides ranked by support vector machine regression. Vertical small bars represent ± 2 times the standard deviation of the average values. msScores are *exactMatchScore_I* and *matchScore_I*. In group 1, non-MS scores were *mutAffinity, mutPeptideStability*, and *rnaExpr*. In group 2, the non-MS scores were the scores from group 1 plus *wtAffinity* and *wtPeptideStability* that take into consideration also aspects related to the wt peptide counterparts. In group 3, non-MS scores were the scores from group 1 plus *diffAffinity, diffPeptideStability*, which incorporate the differences in the binding affinity and in the binding stability, respectively, of the wt and the mutant peptides. *diffAffinity* = *mutAffinity*−*wtAffinity* and *diffPeptideStability* = *mutPeptideStability*−*wtPeptideStability*.

We propose that large-scale and in-depth peptidomics MS/MS data of naturally presented ligands can be used to complement existing predictors and to improve HLA-I neoantigen prioritization. We test this concept using the Stronen et al. data (see Materials and Methods for more details). To develop this approach, we first defined three immunopeptidomics MS-based features. The first feature, at the protein level, is the number of HLA-I peptides per protein in our database (*nrMatchingPeptides_I*). We anticipate that proteins that are highly presented in their wild-type form will have a better chance to present neoantigens once they are mutated. On the peptide level, a match between a predicted peptide and our database can be *exact, included* or *partial* (Figure 5A). We expect that if the mutation is in a position in the source protein that is naturally presented, then we have evidence that the neoantigens could also be presented. Obviously, this will not hold if the mutation falls on a HLA-binding site or otherwise strongly weakens HLA binding, but for this proof of concept we will not consider these events. We added two additional features that quantify the overlap of the predicted wild-type (wt) -peptide with other HLA-I peptides in our database, *exactMatchScore_I* and *matchScore_I* (Table S3 in Supplementary Material). Please refer to Section Materials and Methods for a more detailed description of these scores. These scores reflect the propensity of the wt-peptide to be presented as a HLA-I peptide. As a simplification, we assume that a mutation does not completely obfuscate this propensity, just as the wt-affinity is still a good predictor for the mutated peptide affinity and immunogenicity.

Applying all predictors to the data published by Stronen et al. (see Materials and Methods), we show that the MS-based features provide valuable information for the prioritization of the neoantigens (Figure 5B). The performance of a single MS-based feature was slightly better than the RNA abundance feature, but lower than the affinity and stability features. Overall, 16 out of 1,034 peptides were immunogenic (1.5%), whereas 3 out of 66 peptides with a positive *matchScore I* (4.5%) and 1 out of 7 (14.2%) with a positive *exactMatchScore I* were immunogenic, i.e., the MS-based scores are able to enrich immunogenic peptides. One striking observation was that all 16 immunogenic peptides belonged to proteins that were present in *ipMSDB*, which is unlikely to happen by chance (872 out of all 1,034 peptides did match *ipMSDB*, which corresponds to a probability of 0.065 = (872/1,034)^16^ that the 16 immunogenic peptides are present in *ipMSDB* by chance). Therefore, we improve prediction based on binding affinity of mutated peptides if we remove all predicted sequences which do not belong to any protein in our database. Figure 5C shows how many immunogenic peptides are correctly predicted as a function of the rank of the *mutAffinity* score before (gray line) and after such removal (red line).

Furthermore, we used the Stronen et al. data and the cross-validation scheme outlined in Section Materials and Methods to evaluate how much predictive power the *ipMSDB* features add in combination with non-MS features. We trained a SVM regression on the set of training peptides in order to rank the test peptides according to their SVM-predicted immunogenicity. We then calculated the number of correctly predicted peptides within the 20 top-ranked peptides (see Materials and Methods for more details). We examined how the incorporation of the MS-based features into the SVM prediction improves the number of correctly predicted peptides. When the non-MS scores were *mutAffinity, mutPeptideStability*, and *rnaExpr*, we improved the prediction by 15.9% (group 1). 38.3% prediction improvement was obtained when the non-MS scores were the scores from group 1 plus *wtAffinity* and *wtPeptideStability* (group 2)*. The best improvement of* 50.9% was obtained when the non-MS scores were the scores from group 1 plus *diffAffinity, diffPeptideStability*, which incorporate the differences in the binding affinity and in the binding stability, respectively, of the wt and the mutant peptides (Group 3; where *diffAffinity* is *mutAffinity-wtAffinity* and *diffPeptideStability is mutPeptideStability-wtPeptideStability*) in Figure 5D. These results indicate that our *ipMSDB* based features are complementary to the affinity based features.

Interesting interactions between the MS-based features and affinity-based features emerged, but given the small size of the dataset the following interpretations are speculative. Figure S9 in Supplementary Material shows that almost all immunogenic peptides (blue dots) had a low *mutAffinity* score (low values are good), and that MS-based features were able to rescue the two peptides with the higher *mutAffinity* value (larger orange diamond and red square). Peptides with an *exact* match to the immunopeptidomics MS database (red points) lied close to the diagonal, i.e., had similar *mutAffinity and wtAffinity* scores in which the mutation does not seem to change HLA binding. On the other hand, *included* or *partial* matches can also identify peptides which had different affinities in their mutational and wild type state. In order to confirm these interactions, refine them or find new ones, many more datasets will be required. Also, more work is needed to extend the prediction to peptides, which are often missed by MS/MS analysis such as very hydrophobic peptides or peptides that fragment very poorly.

One of the advantages of our approach is that it may mitigate the limitation of predicting binding affinity for very rare HLA-I alleles and for HLA-II molecules. We deduced information from hundreds of thousands of ligands detected in tens of different individual donors, representing the overall distribution of HLA allotypes in the human population. As the binding specificities of different HLA allotypes may be redundant, in the future, an exhaustive database will provide a good approximation to the definite immunopeptidome. As we know from many studies, immunogenicity of peptides is not highly correlated with binding affinity (26, 58, 59), and peptides with (measured) low affinity may still induce an immune response, especially upon vaccination. However, the peptides should still be naturally presented on the target cells to induce an effective T-cell response. We envision that the pan-HLA-peptide interaction predictors will provide estimation of the binding affinity, and together with an exhaustive immunopeptidomics database, the prioritization of neoantigens will include hotspot features, which capture other aspects of the natural *in vivo* presentation.

In this preliminary, proof of concept study we showed how immunopeptidomics database comprising many melanoma tissue samples contained information that enabled prioritization of neoantigens predicted in similar melanoma samples. We anticipate that the same tumor type will have to be adequately represented in the database to overcome and reflect tissue specific expression signatures. The scoring scheme we introduced here may already be implemented providing large in-depth immunopeptidomics data matching the investigated tumor is present. Yet, much more data from T cell based assays of both immunogenic and confirmed non immunogenic neoantigens from multiple patients across different tumor types will be critical to sorely benchmark and optimize this algorithm.

## Conclusion

Given the rise in the number of research labs performing large-scale immunopeptidomics and the growing interest in detecting neoantigens by MS, it is very likely that within the coming years, comprehensive databases of naturally presented immunopeptidomes from thousands of donors and HLA allotypes will be characterized. This will inevitably lead to a deeper understanding not only of the binding specificities of each of the HLA molecules (28, 29, 32–34) but also of the rules governing sampling of proteins and of the cellular machineries that are involved, in a cell type specific manner (28). The immunopeptidomics data do not contribute to the understanding of immunogenicity seen from a tolerance perspective, since they do not provide information about which neoepitopes are sufficiently “foreign” to induce a T cell response. Therefore, high-throughput functional T-cell screening assays will be fundamental in resolving the propensity of these presented peptides to induce the CD8^+^ and CD4^+^ immune response (20). We envision that combining improved HLA-binding predictions together with information about *in vivo* presentation and their recognition by effector T-cells will significantly improve the accuracy of neoantigen prediction algorithms. Consequently, more patients could benefit from the promising personalized neoantigen-based treatments.

## Ethics Statement

This study was carried out in accordance with the recommendations of the institutional review board [Ethics Commission, University Hospital of Lausanne (CHUV)] with written informed consent from all subjects. All subjects gave written informed consent in accordance with the Declaration of Helsinki. The protocol was approved by the Ethics Commission, University Hospital of Lausanne (CHUV).

## Author Contributions

Conception and design of the work, analysis and interpretation of data, manuscript writing: MM and MB-S; critical revision for important intellectual content: DG and GC. All authors approved the final version of the manuscript and agreed to be accountable for all aspects of the work in ensuring that questions related to the accuracy or integrity of any part of the work are appropriately investigated and resolved.

## Conflict of Interest Statement

The authors declare that the research was conducted in the absence of any commercial or financial relationships that could be construed as a potential conflict of interest.

## Footnotes

^1^www.java.com.

^2^www.r-project.org.

^3^http://www.pantherdb.org/.

^4^www.proteomaps.net.

^5^http://www.cbs.dtu.dk/services/NetMHCpan/.
