# Supplementary material for: ‘Hotspots’ of Antigen Presentation Revealed by Human Leukocyte Antigen Ligandomics for Neoantigen Prioritization
Source: Front Immunol. 2017 Oct 20;8:1367. doi: 10.3389/fimmu.2017.01367 (PMC5654951; doi:10.3389/fimmu.2017.01367)
Supplement: Supplementary file 1 [file Table_1.DOCX]

Supplementary Table 1: List of samples included in our database.

| Sample | Origin | Data source |
| --- | --- | --- |
| Apher1_I | Leukapheresis | 10.1101/098780 |
| Apher6_I | Leukapheresis | 10.1101/098780 |
| BP455_II | B cell | Not yet published |
| CD165_I | B cell | 10.1101/098780 |
| CD165_II | B cell | Not yet published |
| CM467_I | B cell | 10.1101/098780 |
| GD149_I | B cell | 10.1101/098780 |
| GD149_II | B cell | Not yet published |
| H1650_I | ATCC | Not yet published |
| HCC1143_I | ATCC | 10.1074/mcp.M114.042812 |
| HCC1937_I | ATCC | 10.1074/mcp.M114.042812 |
| HCC2218_I | ATCC | Not yet published |
| HCT116_I | ATCC | 10.1074/mcp.M114.042812 |
| HepG2_I | ATCC | 10.1053/j.gastro.2015.05.055 |
| HMEC1_I | ATCC | Not yet published |
| HMEC2_I | ATCC | Not yet published |
| JY_I | B cell | 10.1074/mcp.M114.042812 |
| JY_II | B cell | Not yet published |
| MCF7_I | ATCC | Not yet published |
| MD155_I | B cell | 10.1101/098780 |
| MDA_I | ATCC | Not yet published |
| Mel-12_I | Melanoma tissue | 10.1038/ncomms13404 |
| Mel-15_I | Melanoma tissue | 10.1038/ncomms13404 |
| Mel-15_II | Melanoma tissue | 10.1038/ncomms13404 |
| Mel-16_I | Melanoma tissue | 10.1038/ncomms13404 |
| Mel-16_II | Melanoma tissue | 10.1038/ncomms13404 |
| Mel-20_I | Melanoma tissue | 10.1038/ncomms13404 |
| Mel-21_I | Melanoma tissue | 10.1038/ncomms13404 |
| Mel-21_II | Melanoma tissue | 10.1038/ncomms13404 |
| Mel-24_I | Melanoma tissue | 10.1038/ncomms13404 |
| Mel-24_II | Melanoma tissue | 10.1038/ncomms13404 |
| Mel-25_I | Melanoma tissue | 10.1038/ncomms13404 |
| Mel-25_II | Melanoma tissue | 10.1038/ncomms13404 |
| Mel-26_I | Melanoma tissue | 10.1038/ncomms13404 |
| Mel-27_I | Melanoma tissue | 10.1038/ncomms13404 |
| Mel-28_I | Melanoma tissue | 10.1038/ncomms13404 |
| Mel-28_II | Melanoma tissue | 10.1038/ncomms13404 |
| Mel-29_I | Melanoma tissue | 10.1038/ncomms13404 |
| Mel-29_II | Melanoma tissue | 10.1038/ncomms13404 |
| Mel-3_I | Melanoma tissue | 10.1038/ncomms13404 |
| Mel-30-I | Melanoma tissue | 10.1038/ncomms13404 |
| Mel-33_I | Melanoma tissue | 10.1038/ncomms13404 |
| Mel-34_I | Melanoma tissue | 10.1038/ncomms13404 |
| Mel-35_I | Melanoma tissue | 10.1038/ncomms13404 |
| Mel-36_I | Melanoma tissue | 10.1038/ncomms13404 |
| Mel-38_I | Melanoma tissue | 10.1038/ncomms13404 |
| Mel-38_II | Melanoma tissue | 10.1038/ncomms13404 |
| Mel-39_I | Melanoma tissue | 10.1038/ncomms13404 |
| Mel-39_II | Melanoma tissue | 10.1038/ncomms13404 |
| Mel-4_I | Melanoma tissue | 10.1038/ncomms13404 |
| Mel-40_I | Melanoma tissue | 10.1038/ncomms13404 |
| Mel-41_I | Melanoma tissue | 10.1038/ncomms13404 |
| Mel-41_II | Melanoma tissue | 10.1038/ncomms13404 |
| Mel-42_I | Melanoma tissue | 10.1038/ncomms13404 |
| Mel-5_I | Melanoma tissue | 10.1038/ncomms13404 |
| Mel-8_I | Melanoma tissue | 10.1038/ncomms13404 |
| O07z_I | Ovarian cancer tissue | Not yet published |
| O73C_I | Ovarian cancer tissue | Not yet published |
| PD42_I | B cell | 10.1101/098780 |
| PD42_II | B cell | Not yet published |
| RA957_I | B cell | 10.1101/098780 |
| RA957_II | B cell | Not yet published |
| SupB15_I | ATCC | 10.1074/mcp.M114.042812 |
| TIL1_I | T cell REP | 10.1101/098780 |
| TIL1_II | T cell REP | Not yet published |
| TIL3_I | T cell REP | 10.1101/098780 |
| TIL3_II | T cell REP | Not yet published |
