# Supplementary material for: ‘Hotspots’ of Antigen Presentation Revealed by Human Leukocyte Antigen Ligandomics for Neoantigen Prioritization
Source: Front Immunol. 2017 Oct 20;8:1367. doi: 10.3389/fimmu.2017.01367 (PMC5654951; doi:10.3389/fimmu.2017.01367)
Supplement: Supplementary file 4 [file Data_Sheet_1.ZIP › Legends Supplementary Figures 2,4,5.docx]

# ‘Hotspots’ of Antigen Presentation Revealed by HLA Ligandomics For Neoantigen Prioritization

Müller et al.

Supplementary Figure 2: Detailed high resolution Proteomaps visualization of the level of presentation (number of unique peptide sequences) of “type I” source proteins classified according to their annotated cellular functions.

Supplementary Figure 4: Detailed high resolution Proteomaps visualization of the level of presentation (number of unique peptide sequences) of “type II” source proteins classified according to their annotated cellular functions.

Supplementary Figure 5: Detailed high resolution Proteomaps visualization of the level of presentation (number of unique peptide sequences) of “type I/II” source proteins classified according to their annotated cellular functions.
