# Supplementary figures and images for: ‘Hotspots’ of Antigen Presentation Revealed by Human Leukocyte Antigen Ligandomics for Neoantigen Prioritization
Source: Front Immunol. 2017 Oct 20;8:1367. doi: 10.3389/fimmu.2017.01367 (PMC5654951; doi:10.3389/fimmu.2017.01367)

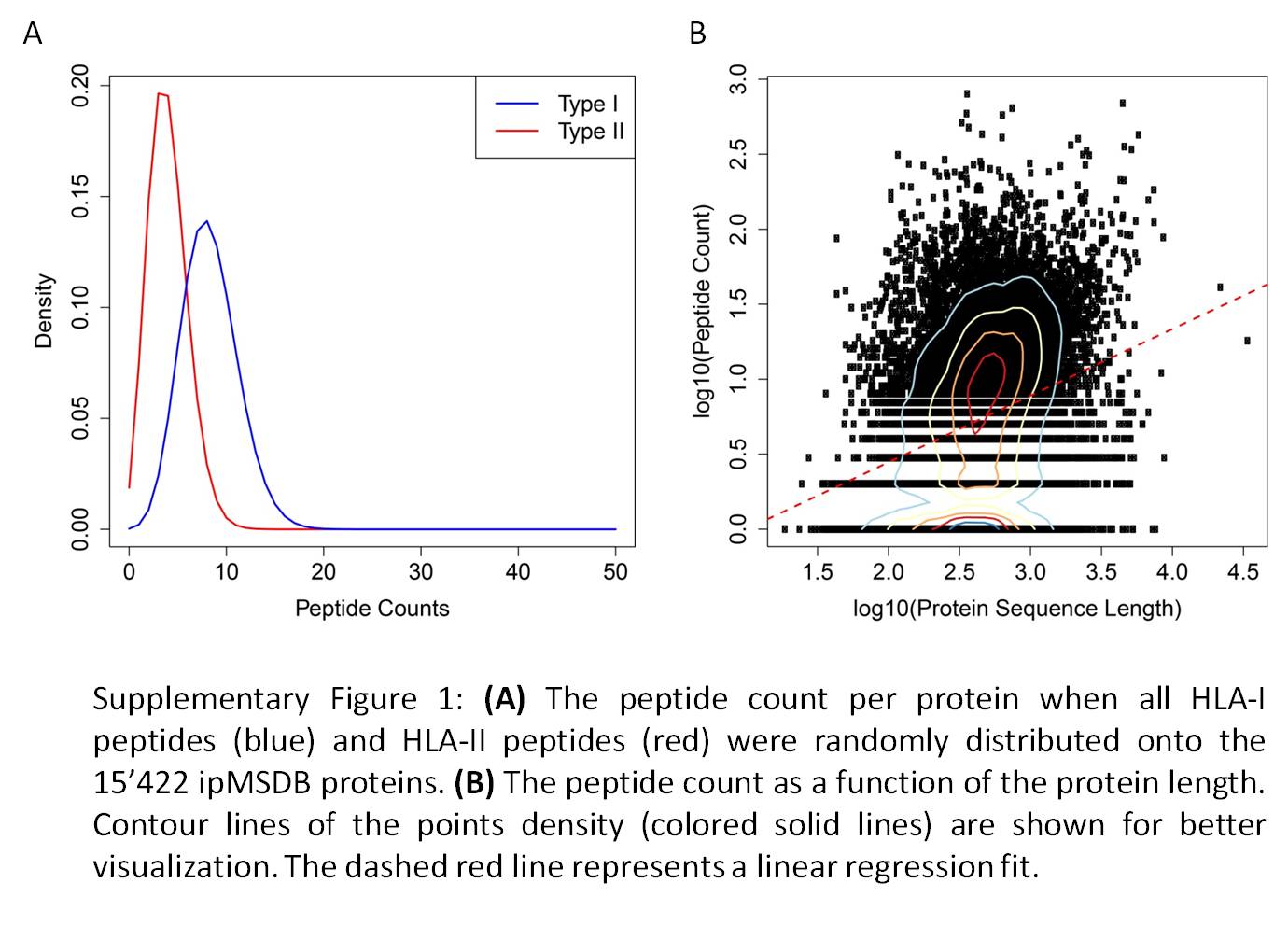

Supplement: Supplementary file 5 [file Image_1.JPEG]

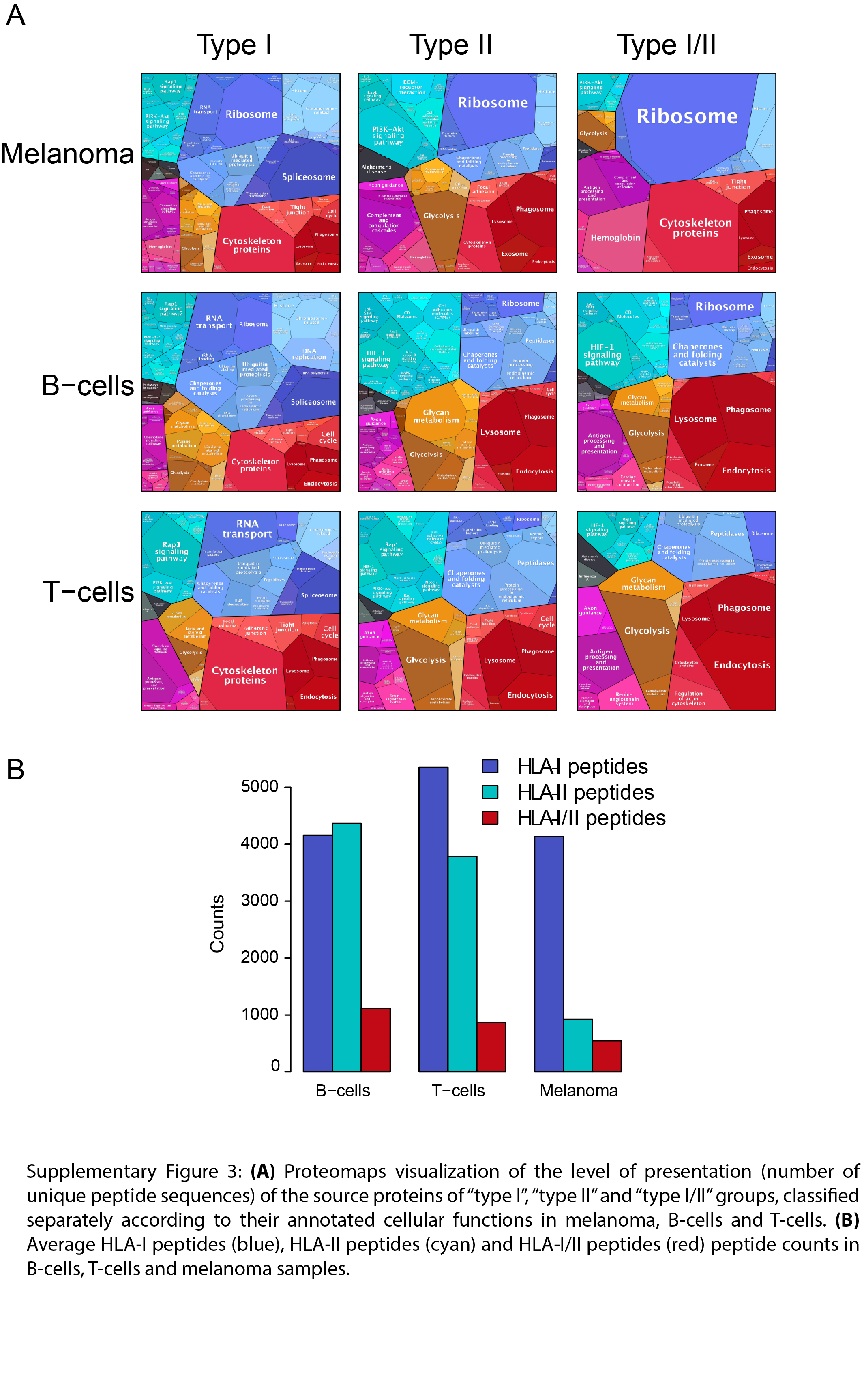

Supplement: Supplementary file 6 [file Image_3.JPEG]

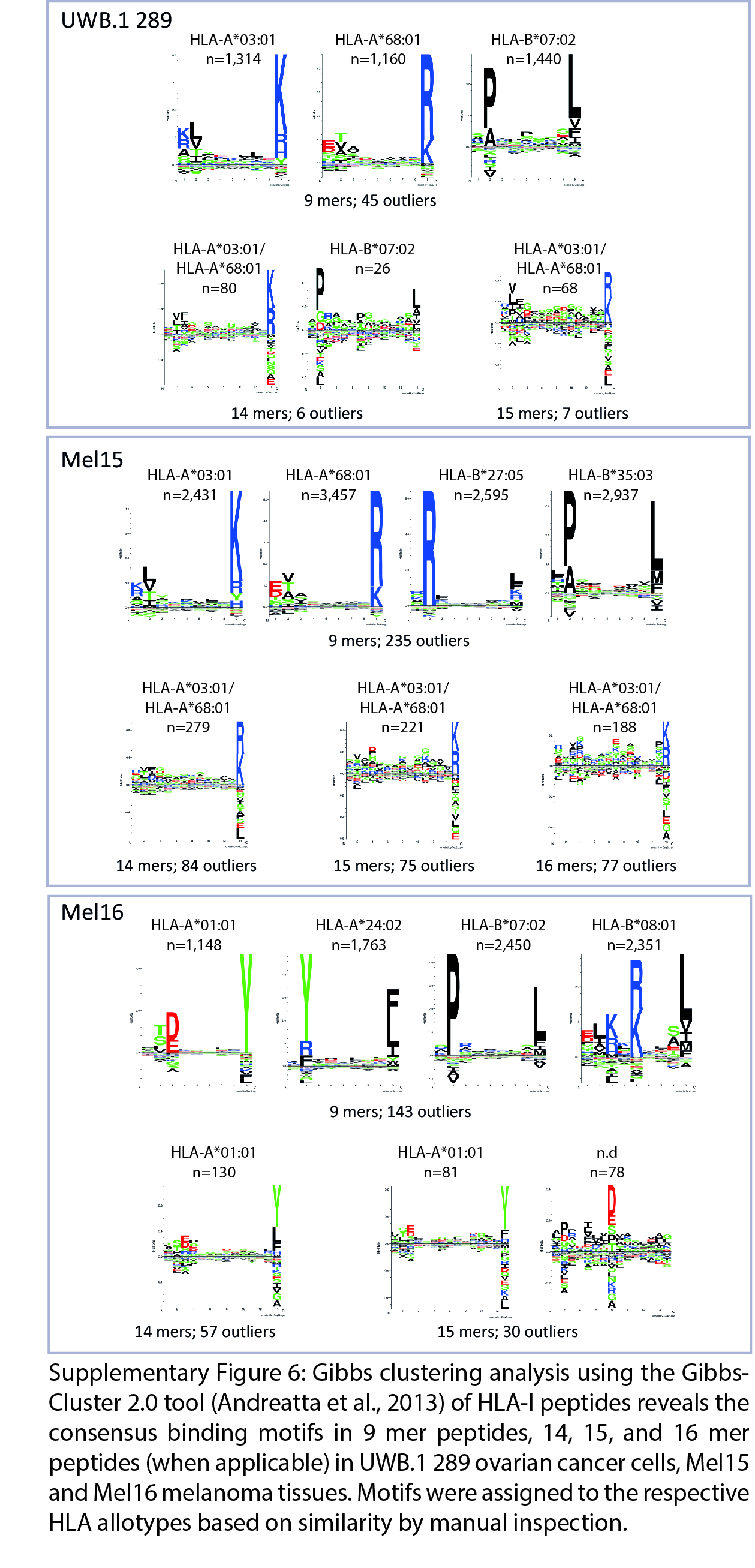

Supplement: Supplementary file 7 [file Image_6.JPEG]

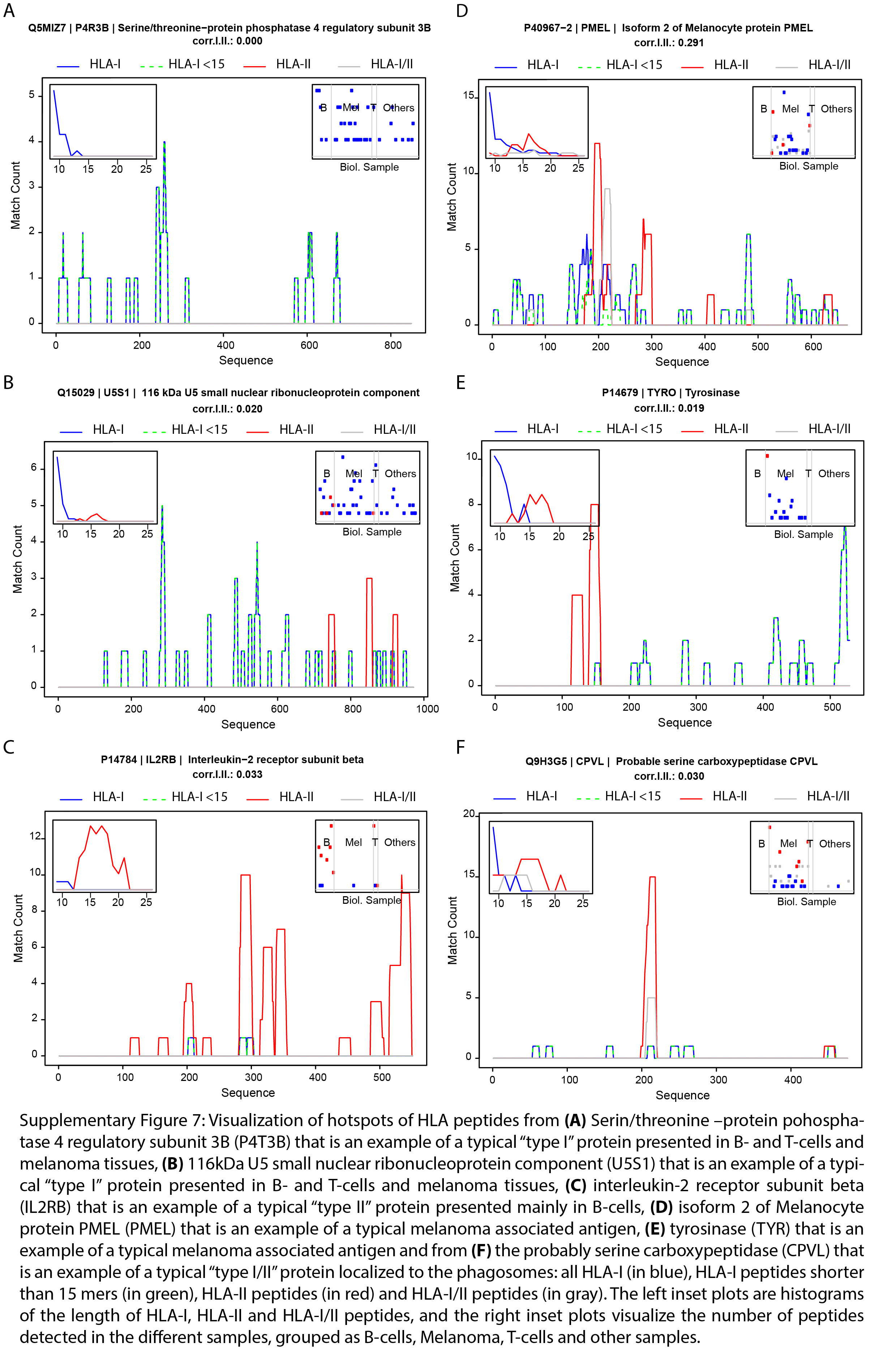

Supplement: Supplementary file 8 [file Image_7.JPEG]

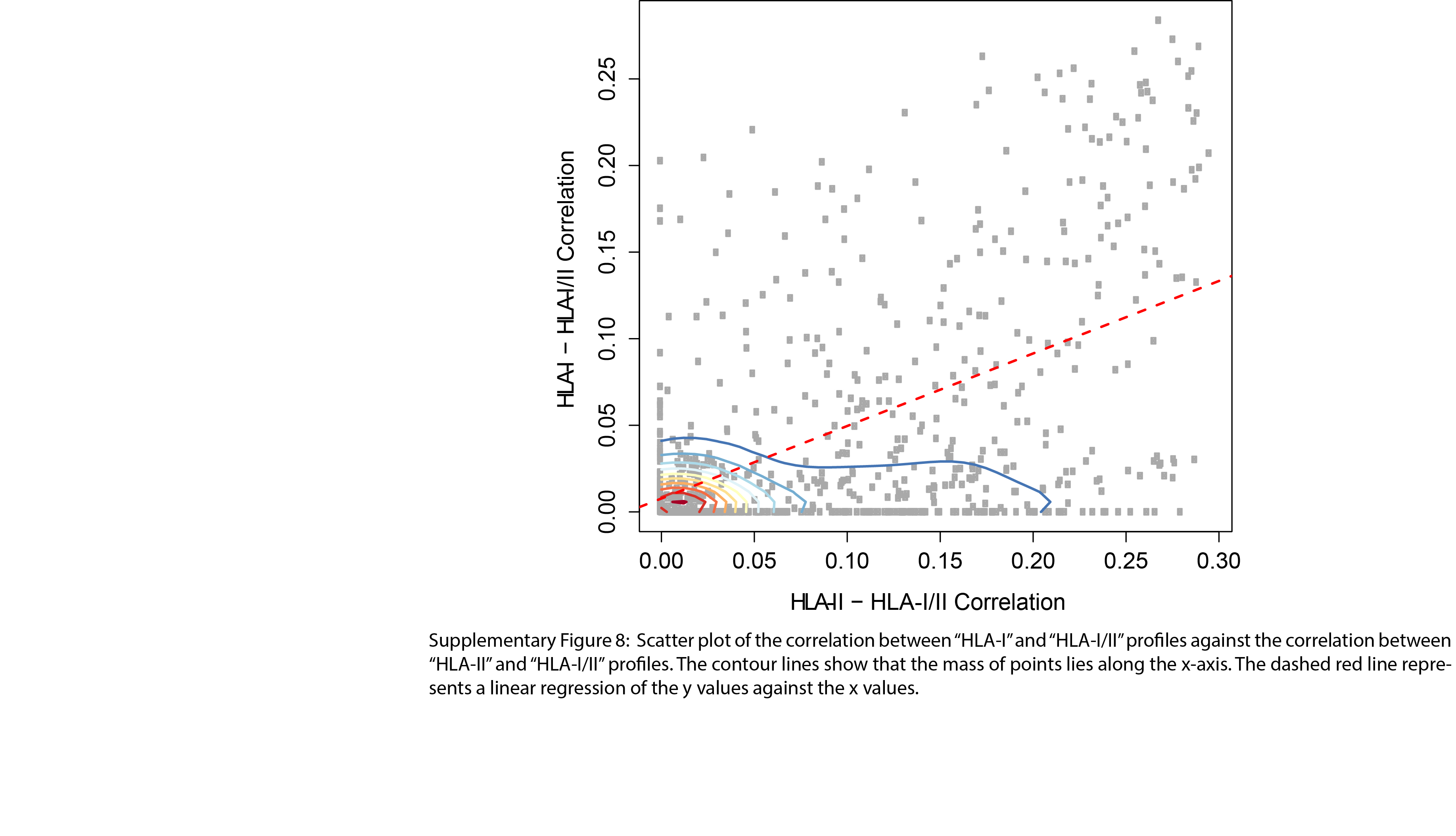

Supplement: Supplementary file 9 [file Image_8.JPEG]

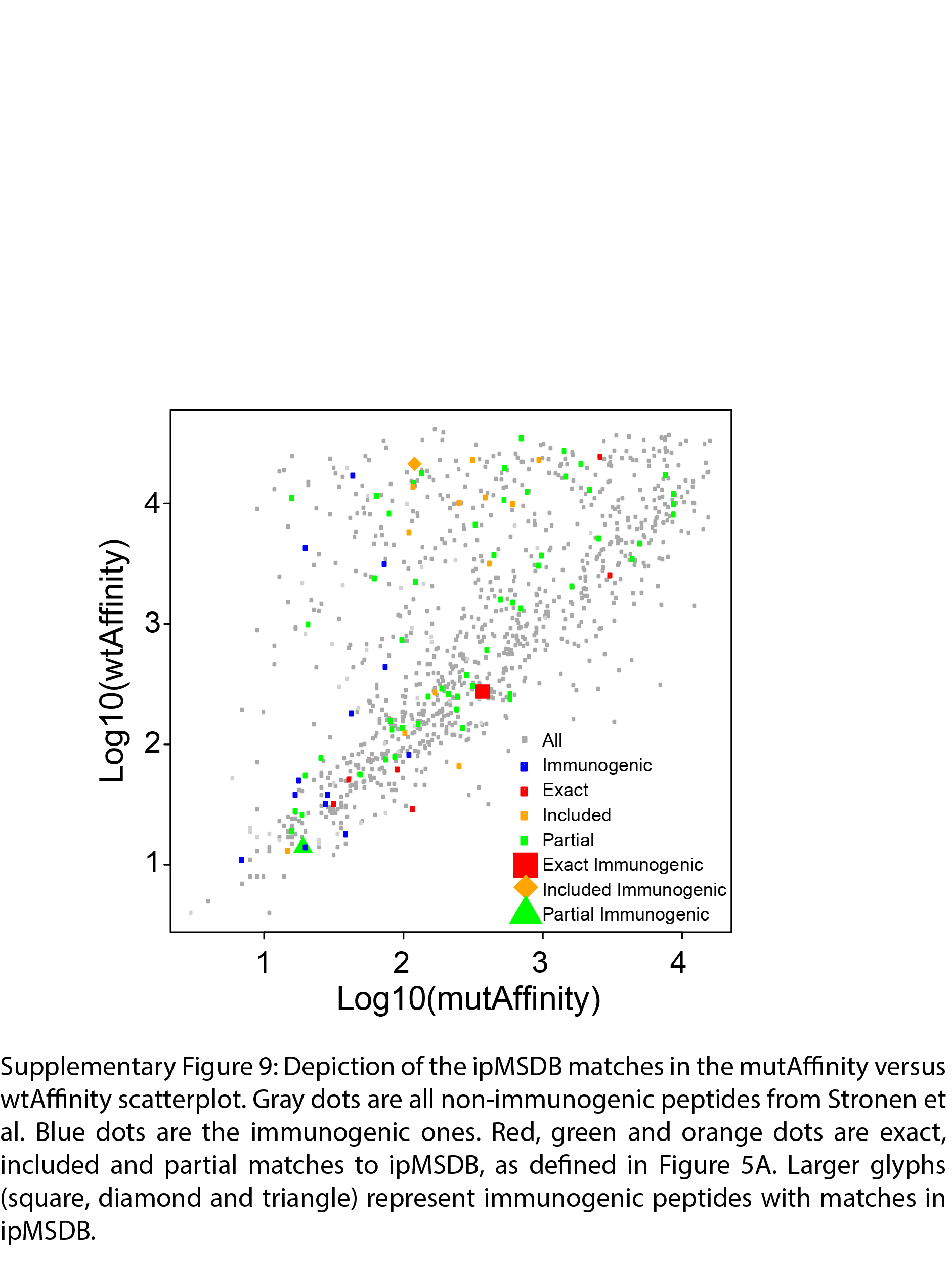

Supplement: Supplementary file 10 [file Image_9.jpg]
